# Factor VII Deficiency: Clinical Phenotype, Genotype and Therapy

**DOI:** 10.3390/jcm6040038

**Published:** 2017-03-28

**Authors:** Mariasanta Napolitano, Sergio Siragusa, Guglielmo Mariani

**Affiliations:** 1 Hematology Unit-Reference Regional Center for Thrombosis and Hemostasis, Università di Palermo, 90127 Palermo, Italy; sergio.siragusa@unipa.it; 2 Westminster University, London W1B 2HW, UK; g.mariani@westminster.ac.uk

**Keywords:** inherited Factor VII deficiency, diagnosis, bleeding, replacement therapy, genotype

## Abstract

Factor VII deficiency is the most common among rare inherited autosomal recessive bleeding disorders, and is a chameleon disease due to the lack of a direct correlation between plasma levels of coagulation Factor VII and bleeding manifestations. Clinical phenotypes range from asymptomatic condition—even in homozygous subjects—to severe life-threatening bleedings (central nervous system, gastrointestinal bleeding). Prediction of bleeding risk is thus based on multiple parameters that challenge disease management. Spontaneous or surgical bleedings require accurate treatment schedules, and patients at high risk of severe hemorrhages may need prophylaxis from childhood onwards. The aim of the current review is to depict an updated summary of clinical phenotype, laboratory diagnosis, and treatment of inherited Factor VII deficiency.

## 1. Introduction

Inherited Factor VII deficiency is the most common among rare autosomal recessive bleeding disorders. Coagulation Factor VII (FVII) is a plasma vitamin K-dependent serine protease produced by the liver. Factor VII is the unique coagulation factor comprising with a small proportion (1%–3%) of free circulating activated form (FVIIa), even in the absence of coagulation activation [1]. Blood clotting is initiated by the interaction between tissue factor (TF) exposed on the vascular lumen upon injury and FVIIa [2]. The FVIIa–TF complex is able to activate factors IX (to FIXa) and X (to FXa), which ultimately induce the formation of a stable ﬁbrin clot [3]. All domains of FVIIa are involved in the interaction between TF and FVIIa: the catalytic domain, the gamma-carboxyglutamic acid domain, and the two epidermal growth factor (EGF)-like domains [4]. Only small amounts of FVIIa are needed to trigger coagulation. Inherited Factor VII deficiency is characterized by a wide spectrum of clinical phenotypes (Table 1) ranging from asymptomatic condition—even in homozygous subjects—to severe life-threatening bleedings including central nervous system (CNS) and gastrointestinal (GI) bleeding [5,6,7]. Studies on knockout mice indicate that the absence of FVII is incompatible with life; thus, FVII deﬁciency could not be associated with a complete lack of functional FVII [8,9]. FVII plasma levels can be influenced by FVII gene polymorphisms [10]. Several studies on the molecular structure and genetic diagnosis of FVII have been performed [7,11,12,13,14]. Due to the rarity of the disease, the optimal definition of clinical symptoms and treatment has been reached mainly thanks to international multicenter studies like the International Registry on Congenital Factor VII Deficiency (IRF7) [5] and the Seven Treatment Evaluation Registry (STER) [13]. 

## 2. Clinical Manifestations

Inherited FVII deficiency has a wide range of clinical manifestations; mild (muco-cutaneous) bleeds (like in platelet disorders) are the most frequent, while 10% to 15% exhibit potentially life- or limb-threatening hemorrhages (CNS, GI, or hemarthrosis) [1,6,14]. About one-third of patients with FVII deficiency tend to remain asymptomatic during their life [5]; these subjects are diagnosed as part of family studies or hemostatic screenings. The most frequently reported bleeding symptoms among “platelet-like” FVII deficiency are epistaxis (60%), gum bleeding (34%), easy bruising (36%), and menorrhagia (69% of females) [5]. These manifestations usually do not require any systemic replacement therapy and are well-controlled by local hemostatic agents [5,15], even if in some cases (i.p. menorrhagia) treatment and prophylaxis may be required [16]. Excessive menstrual blood losses have been objectively determined in women with inherited Factor VII deficiency [17]; they affect two thirds of patients of fertile age, with a negative impact on quality of life [18]. Women with Factor VII deficiency can suffer from ovarian cysts and ovulatory bleeding; they also have an increased risk of post-partum hemorrhage [19,20].

Among the severe forms, recurrent hemarthrosis (19%) and gastrointestinal bleeding are relatively frequent (15%); central nervous system bleeding is less frequent (2.5%) but it can be the most serious and life threatening symptom [5]. Severe bleedings usually occur soon after birth or in infants and bleeding symptoms follow the same specific pattern of their onset. In detail, umbilical cord stump bleeds are associated with a high risk of developing further severe bleeds (CNS and GI) at a very young age [14]. Intracranial hemorrhage (ICH) may be the presenting symptom in a newborn. Bleeding after circumcision or heel stick during the neonatal period and dental extractions in childhood is quite common in inherited Factor VII deficiency [21]. There is a lack of correlation between clinical phenotypes and FVII coagulant activity (FVII:c). It is not rare to observe asymptomatic subjects with FVII:c below 1%, or on the other hand, patients with FVII:c level above 5% with a history of severe hemorrhages [1,6]. Heterozygous patients usually do not experience severe symptoms [22]. Minimal levels of FVII:c able to protect from bleeding in different clinical scenarios are also not well defined, ranging from 8% [23] to 15%–20% [24]. It is generally accepted that FVII:c levels above 20% can protect from spontaneous bleedings [6,25], and data from IRF7 and STER have shown a minimal clinical relevance for FVII:c below 26% [14]. This unpredictable behavior could be related to the combined enhanced effect of TF–FVIIa complex, able to activate coagulation even with small amounts of FVII [4]. However, the risk of bleeding in asymptomatic individuals cannot be underestimated in circumstantial situations like major surgery [20,26]. Each bleeding type increases the likelihood of an early diagnosis of Factor VII deficiency, and asymptomatic individuals at diagnosis may experience bleeding, if any, late in life [14].

## 3. Diagnosis

Inherited Factor VII deficiency is usually diagnosed with a laboratory workup performed after a bleeding episode or during familial screening in cases with a known history of the disease. Factor VII deficiency can also be a serendipitous finding. Basic diagnostic work-up includes routine assays (prothrombin time, PT, activated partial thromboplastin time, aPTT and platelet count) followed by FVII coagulant activity measurement for isolated prolonged PT, corrected by mixing studies. To confirm the diagnosis, FVII assay should be repeated at least once. The assay of the other vitamin K-dependent clotting factors is not necessary but useful to rule out the combined deficiencies of vitamin K-dependent factors in which case, however, the aPTT is also prolonged [27].

FVII deficiency is usually distinguished into two types: type I (quantitative defects, with decreased FVII:c and FVII antigen, FVII:Ag), type II (qualitative defects, with low FVII:c and normal FVII:Ag). FVII coagulant activity is determined with a one-stage assay mixing citrated plasma sample, FVII-deficient reagent, and thromboplastin [28]. The accuracy of FVII:c assay is related to the sensitivity of the thromboplastin used, calibrators, and the quality of the FVII:c-deficient reagent [29]. Thromboplastins contaminated with small amounts of FVIIa may activate plasma and decrease the sensitivity to FVII:c assay [30]. This is particularly important for the diagnosis of type II defects, like FVII Padua. The Padua variant of FVII deficiency (Arg304Gln in exon 8) shows a different reactivity toward different tissue thromboplastins and determines normal results when brain thromboplastin is employed [31]. On the contrary, type I defect has never been reported to display variable reactivity toward different thromboplastins [32].

FVII antigen levels can be determined by enzyme-linked immunosorbent assays or immune-turbidimetric assays, with monoclonal epitope-specific antibodies. FVII:Ag assay allows distinguishing between type I and II defects. Furthermore, FVII:Ag does not allow the prediction of bleeding tendency, but it may contribute to clarifying the role of specific mutational mechanisms of the defect.

FVIIa assays have been adopted to monitor the treatment with recombinant activated FVII (rFVIIa) in patients with FVII-deficiency and in hemophilia with inhibitors. The method adopts recombinant TF, acting as a cofactor for FVIIa but unable to activate FVII [33]. New, chromogenic or fluorogenic assay methods for FVIIa are now available for research purposes. FVIIa assay—even if more effective in monitoring treatment with rFVIIa than FVII:c assay [34]—is not recommended in the diagnosis of FVII deficiency.

## 4. Genetic Diagnosis

Correlation between genotype–phenotype indicates that modifiers (environmental and/or inherited factors) modulate the expressivity of FVII deficiency, as mirrored by patients with the same gene mutation and discordant clinical phenotypes. FVII gene mutations are heterogeneous. 

The FVII gene was sequenced for the first time in 1987; the whole sequence has been widely studied by DNA sequencing. Nowadays, direct gene sequencing allows detection of the mutation in 90%–92% alleles. Whole gene sequencing is the recommended method for molecular diagnosis of FVII due to a high frequency of single nucleotide (point) mutations, a low recurrence of the same mutations, and a short length of the gene. In up to 10% of FVII deficiencies, a gene mutation has not been identified by DNA sequencing. This could be related to mutations in a different gene [9]. To further understand clinical variation among individuals with the same gene mutation, studies have investigated the issue of clinical picture modifiers (i.e., common thrombophilia markers) not reaching firm conclusions [35]. Tissue factor, von Willebrand factor, or platelets could also be involved in the expression of bleeding tendency in FVII deficiency. Unknown regulators are not excluded either [20]. 

Prenatal diagnosis—in particular genetic analysis of cord blood [36]—is recommended for families with a history of severe or life-threatening bleeding episodes. FVII:c could also be assayed on cord blood obtained after cordocentesis between the 17th and 21st weeks of gestation; however, in this last case a confirmation test is needed after birth [37].

Genetic testing is not routinely adopted for the diagnosis of FVII deficiency, unless prenatal diagnosis is required.

## 5. Replacement Therapy

Bleeding risk prediction and treatment options for acute spontaneous bleeding episodes or surgery are challenging. FVII:c levels alone cannot drive the management of the disease, as they are not able to accurately predict the bleeding risk tendency and currently, guidelines for the management of this rare bleeding disorder are lacking. For replacement therapy, the analysis of a number of factors is required, such as the rarity of the disorder, the type, availability and supply of products, as well as economic and geographical factors.

Results from multicenter observational studies like STER and IRF7 have provided important elements of evidence related to the efficacy and safety of replacement therapy (RT) as well as the schedules for spontaneous bleeding episodes [15], major surgery [38], invasive procedures [39] and prophylaxis [16]. These trials have contributed to prediction of the bleeding risk of patients with inherited FVII deficiency mainly based on the first bleeding symptom, the personal clinical history, and FVII:c levels [14]. Several therapeutic options are currently available for the treatment of inherited FVII deficiency: i. recombinant activated Factor VII, ii. plasma-derived Factor VII, iii. fresh frozen plasma and iv. prothrombin complex concentrates. 

Recombinant FVIIa (rFVIIa) is the most used replacement therapy, when available. Pharmacokinetic studies in patients with inherited Factor VII deficiency at steady state have shown a large volume of distribution and a prolonged pharmacodynamics effect of the drug [40].

Data from STER (Table 2) have shown that one day RT with “intermediate” doses (median 60 μg/kg) of rFVIIa [15] can be effective and safe for the treatment of bleeds like hemarthrosis, muscle/subcutaneous hematomas, epistaxis, and gum bleeding. Single- or multiple-dose schedules can be used for the treatment of menorrhagia. Short- or long-term prophylaxis is indeed warranted for the patients with the most severe bleeding picture (CNS, GI) [16]. Heavy menstrual bleeding in women with inherited bleeding disorders should be managed with a multidisciplinary approach, based also on the objective of preserving fertility [41]. Other medical therapies of menorrhagia includes hemostatic agents like tranexamic acid and hormonal treatments (combined oral contraceptives, levonorgestrel intrauterine devices), in combination or not with RT [42]. Progesterone-releasing intra-uterine systems can be effective in suppressing ovulation, thereby reducing the risk of bleeding in these women. RT alone may be difficult to accept—in particular for young women after menarche [43]. 

In the management of major surgical procedures (particularly during the first 24 h after the procedure), replacement therapy with rFVIIa is effective if administered at a minimum dose of 13 μg/kg/body weight per single dose, in at least three administrations [38]. For invasive procedures and minor surgery, 1-day RT resulted sufficient, with an “average” total dose of rFVIIa of 20 μg/kg/body weight, possibly in more than one administration [39]. RT with rFVIIa has a very good safety-to-efficacy ratio [38,39,44,45]. Continuous infusion of rFVIIa during surgery has also been reported as effective, safe, and well-tolerated in patients with inherited Factor VII deficiency [46].

Adverse events (AEs)—mainly inhibitors to FVII and thrombosis—reported from STER had a low to very-low incidence [44], with three cases of thrombosis and two cases of confirmed inhibitors among 245 reported treatments with rFVIIa.

Plasma-derived FVII (pd-FVII) concentrates may be an alternative option to rFVIIa. Large volume infusions and the short half-life (4–6 h) of FVII make this administration option less appealing, particularly in children. One IU of pd-FVII induces a plasma level increase of 1.9%. Currently, the average pd-FVII dosages used are 15–20 IU/kg for mucosal bleeding, and 30–40 IU/kg in severe or life-threatening hemorrhages [47].

Fresh frozen plasma (FFP) can be more easily available; however, its effectiveness is limited by a high risk of fluid overload following repeated infusions [48]. FFP is currently given at a mean dose of 15–30 mL/kg depending on the site and severity of bleeding [15].

Antifibrinolytic agents like tranexamic acid—alone for mild bleedings or in combination with RT—can be effective [16,49].

Long-term prophylaxis is indicated in patients with a severe clinical phenotype (CNS and GI bleedings) since childhood and soon after the first bleeding event [16,24]. Data from the STER showed that recombinant activated factor VII prophylaxis schedules based on “frequent” administrations (three times weekly) of 90 μg/kg total weekly dose resulted effective. Short-term or intermittent prophylactic schedules can also be considered, particularly in conditions like menorrhagia or severe hemarthroses [16,20]. Early long-term primary prophylaxis in children with severe FVII deficiency with very high bleeding risk was proved to be safe and effective [50]. 

Plasma-derived FVII concentrates have been prophylactically administered for periods from 9 months to 9 years at dose schedules 10–50 U/kg/three times per week or on alternate days and was shown to be effective in reducing the frequency and/or severity of bleeding [51]. Available evidence does not support a long-term prophylaxis with FFP, due to persistence of significant bleeding in patients on FFP [52].

## 6. Conclusions

We have here described clinical manifestations, diagnosis, and treatment principles in inherited FVII deficiency—a rare but complex bleeding disorder. Available data is mainly based on results stemming from observational studies and case series: the registries, however have provided convincing evidence that this disorder may be managed with success with little burden of adverse events. Treatment, however, is still challenged by product availability worldwide.

## Figures and Tables

**Table 1:** High-risk and low-risk patients with inherited Factor VII deficiency, based on bleeding history and coagulation Factor VII (FVII) activity.

Bleeding	FVII:c (%)	Personal History	Familial History
High risk *	<2	CNS bleeding, UC stamp bleeding, hemarthrosis, GI bleeding	Life-threatening bleeding, death for hemorrhage in first degree relatives
Low risk #	>20	Negative for spontaneous bleeding	Negative for spontaneous bleeding

CNS: Central Nervous System; FVII:c: FVII coagulant; GI: Gastro-intestinal; UC: Umbilical Cord. * Some patients with FVII:c < 1% may be asymptomatic; # Some patients may bleed with FVII:c > 20%.

**Table 2:** Replacement therapy with rFVIIa in inherited Factor VII deficiency (data from STER studies).

Clinical Context	Treatment Days, Median (Range)	Daily Dose rFVIIa, Median (Range)	Single Dose rFVIIa, Median (Range)
Spontaneous bleeding	1 (1–14)	80 (35–210)	60 (10–3600)
Major Surgery	3.5 (1–16)	31.9 (12–120)	18.7 (6.5–87)
Minor Surgery	1 (1–4)	20 (7.2–300)	20 (19–60)
Prophylaxis	Three/two times per week	--	90 (30–120)

Doses are expressed as μg/kg/bw. rFVIIa: recombinant activated Factor VII (Novoseven^®^).
